# SMOKE IT! Promoting a change of opiate consumption pattern - from injecting to inhaling

**DOI:** 10.1186/1477-7517-11-18

**Published:** 2014-06-27

**Authors:** Heino Johann Stöver, Dirk Schäffer

**Affiliations:** 1 Institute for Addiction Research, Frankfurt 60318, Germany; 2 Deutsche AIDS Hilfe e.V., Berlin 10963, Germany

**Keywords:** Drug use, Foil, SMOKE IT, Inhalative, Injection, Harm reduction, Morbidity, Mortality, Route transition interventions

## Abstract

**Background:**

Intravenous drug use has been predominantly practised since illegal heroin use became known in Germany in the early 1970s. The available data suggest that the risk of accidental overdose when smoking heroin is substantially reduced compared to injecting a substance of unknown purity and quality. Moreover, the risk of transmitting HIV, Hepatitis B or C *via* blood contact is considerably reduced when smoking heroin rather than when injecting it intravenously. In spite of the significant strain on the lungs and the respiratory tract caused by smoking, it can be concluded that inhalative use - measured by the indicators ‘overdose’ and ‘viral infections’ is considerably less dangerous than intravenous use. Despite these harm-reducing effects of inhalative use, there is only very limited scientific survey on this subject. The project ‘SMOKE IT!’ studied to what extent a change of the consumption method can be supported by making new equipment for drug use available.

**Method:**

‘SMOKE IT!’ was carried out as a multi-centre survey in drug consumption rooms (DCRs) in five German cities. Participants received ‘SMOKE-IT!’ packs that contained new heroin smoking foils, as well as information about inhalative drug use. The quantitative data collection was aided by a written questionnaire filled out at three different stages in 2012.

**Results:**

The vast majority of the 165 respondents favoured using the foils from the ‘SMOKE-IT!’ packs (82.5%). The survey shows that two-thirds of the sample used the SMOKE-IT foils for inhaling instead of injecting. Almost six out of ten said that smoking was healthier than injecting. Thirty-five percent of the participants named the reduced risk of a hepatitis or HIV infection as a particularly important factor. A third of the respondents used the smoking foils to avoid the danger of an overdose.

**Conclusions:**

Targeted media and personal intervention in association with the dispensation of attractive drug use equipment can motivate opiate users to change their method of drug use. The main reason for inhalative use is that it is significantly less dangerous, measured by the indicators ‘overdose’ and ‘viral infections’. All drop-in centres should expand their syringe-exchange services to include the dispensation of smoking foils.

## Background

Intravenous drug use has been predominantly practised since illegal heroin use became known in Germany in the early 1970s [1-3]. This way of administration is the most hazardous way of using heroin. Due to the bumper crops in the cultivation countries (particularly Afghanistan; see World Drug Report^a^), the price of heroin dropped considerably in Germany and inhalative methods of administration started to be increasingly applied following the turn of the millennium. This is not to be understood as a linear process.

The available data suggest that the risk of accidental overdose when smoking heroin is substantially reduced compared to injecting. Moreover, the risk of HIV and Hepatitis B or C infection is considerably reduced when smoking heroin

Despite these harm-reducing effects of inhalative use, there is only very limited scientific survey on this subject. Kools [4] described the Dutch experience in promoting transition away from injecting drug use to inhaling. Pizzey and Hunt [5,6] studied the introduction of foils in four facilities in the Northwest of England.

As part of the ‘SMOKE-IT!’ project, it is to be investigated to what extent a change in the method of drug use (from intravenous to inhalative) can be promoted by means of new prevention tools as well as media and personal intervention.

Furthermore, it is to be investigated whether the provision of new drug use equipment (foil, tube) as well as accompanying literature (flyers, posters) can promote the willingness to change the method of administration.

This survey is aimed at creating an initial solid basis for investigating the effectiveness of a targeted approach to changing the method of administration by provision of new drug use equipment. Based on these results, targeted campaigns with great infection-preventing benefits could be launched. If the results are positive, the funding organisations will have sufficient reasons to expand their range of harm-reduction services.

## Method

The data was collected using a written questionnaire, which had been designed in cooperation with the staff of the participating drug consumption rooms. A preliminary final version of the questionnaire was pre-tested by the participating facilities in Berlin and Dortmund.

The media (posters, flyers, card) designed, the smoking foils as well as the transparent pouches, which served as a container to hand out all components of the ‘SMOKE-IT!’ pack to the heroin users, were sent over to the participating facilities in April 2012.

The survey was started as scheduled on 1 April 2012.

The questionnaire was to be completed by the interviewed heroin users at three different stages.

The first part of the questionnaire was filled out immediately after recruiting the survey participants (stage T1). It contained questions relating to demographics, duration of opiate use (including the duration of practicing different methods of administration), current heroin use, and the question whether the interviewed persons would like to receive a ‘SMOKE-IT!’ pack.

The respondents who accepted the ‘SMOKE-IT!’ pack were to be interviewed again after using the foil in the consumption room or after returning to the facility (stage T2). The subject of this questionnaire was the use and rating of smoking foils, the reasons for smoking heroin, positive and negative experience with the smoking foils and changes in the method of administration (smoking instead of injecting).

The third and last stage (T3) within this survey was to take place not earlier than 30 days after the survey at T2. The subject of this survey was the use of smoking foils during the last few weeks (since T2), the rating of the foils, (possible) changes in the method of administration and the price participants would be willing to pay to continue using the foils in the future.

A total of six consumption rooms took part in recruiting participants: the ‘SKA’ facility in Berlin (funded by Fixpunkt), the ‘La Strada’ (funded by AH Frankfurt) and ‘Niddastr. 49’ (funded by Integrative Drogenhilfe) facilities in Frankfurt, the ‘Ragazza’ facility in Hamburg (funded by Ragazza), which is run exclusively by women for women, and the KICK facility in Dortmund (funded by AH Dortmund) and Bielefeld (drug counselling centre).

The reason why drug consumption rooms (DCRs; with smoking rooms) were selected for the provision of inhalative material is that the foils could be used in a legal environment and respondents could be reached again more easily for the second and third stages of the survey.

To what extent the proportion of the injecting population might be reached through these services is unclear. There are 24 DCRs in Germany, and the proportion of heroin smoking in most of the facilities, where heroin smoking is allowed, remains unclear. Taking out only Frankfurt with four DCRs and approximately a quarter of a million consumptions were supervised in 2013, only 5% are smoking heroin (Förster and Stöver [7], p. 44).

The survey was based on self-completed questionnaires. If and to what extent the staff was helping the clients in filling in the questionnaires is unclear since the staff was trained not to do so. However, in case the staff did so, this might influence the answers of the respondents. However, the staff members were instructed to just offering the foils among other services. No persuasion was intended, staff just gave it out.

The self-completed questionnaires were collected and were sent over to the evaluating institute (Institute for Addiction Research, ISFF Frankfurt/Main) at a fixed date. After receipt of all questionnaires, the data was recorded using a computer-aided input programme specifically developed for this purpose. The data was subsequently checked for plausibility using the SPSS 15 statistical programme and corrected where necessary. Finally, SPSS was used again to evaluate the data.

The data collection was carried out using an anonymous patient characteristic form which aimed at providing as much confidentiality as possible. The study was voluntary, and all respondents provided their written informed consent.

## Results

### SMOKE IT - highly accepted by drug users in drug consumption rooms

By the end of the quantitative survey (15 August 2012), a total of 177 questionnaires had been received. Twelve persons refused the ‘SMOKE-IT!’ pack. Out of the remaining 165 respondents, 141 were interviewed again at T2. This corresponds to a re-attainment rate of 85.5%. Eighty-nine persons took part in the last survey at T3 (re-attainment rate in relation to T1: 54.0%). During the period of the survey, it was difficult to meet and to offer the questionnaire to participants in the survey for three times (during 4, 5 months). DCRs cannot be understood as utilised on a daily base by most of the people, but rather unfrequently. So it was not possible to meet people three times in the period.

Table 1 lists several characteristics of interviewed heroin users who accepted the offered ‘SMOKE-IT!’ packs. The respective percentages are reported for the stages T1, T2 and T3. This way of presentation allows an estimate of the extent to which drop-outs between the individual stages led to distortions in sampling. In cases where the three samples differ greatly in terms of relevant characteristics; a comparative interpretation of results obtained at different stages would only be possible to a limited extent.

**Table 1:** Characteristics of respondents by stage of interview

	**T1**	**T2**	**T3**
	**%/MV**	**%/MV**	**%/MV**
Place of survey	(*N* = 165)	(*N* = 141)	(*N* = 89)
Berlin	30.9	31.9	20.2
Bielefeld	5.5	6.4	1.1
Dortmund	11.5	11.3	15.7
Frankfurt	46.1	44.7	57.3
Hamburg	6.1	5.7	5.6
Sex	(*N* = 165)	(*N* = 141)	(*N* = 89)
Male	77.0	77.3	79.8
Female	23.0	22.7%	20.2
Age	(*N* = 165)	(*N* = 141)	(*N* = 89)
19–29 years	29.7	30.5%	29.2
30–39 years	40.0	41.8%	46.1
40 years and over	30.3	27.7%	24.7
Average age (SD)	34.7 (8.3)	34.3 (8.4)	34.4 (7.8)
Nationality	(*N* = 161)	(*N* = 138)	(*N* = 88)
Germany	78.9	79.0	81.8
Other EU country	9.3	10.1	8.0
Other country outside the EU	11.8	10.9	10.2
Years of heroin or other opiate use	(*N* = 165)	(*N* = 141)	(*N* = 89)
1–5 years	19.4	19.9	15.7
6–10 years	24.8	27.0	29.2
11–15 years	20.0	17.7	22.5
16–20 years	20.6	21.3	19.1
21 years and over	15.2	14.2	13.5
Mean value (SD)	13.3 (8.3)	13.1 (8.2)	13.2 (8.2)
Years of intravenous heroin use	(*N* = 117)	(*N* = 99)	(*N* = 64)
Mean value (SD)	10.4 (9.0)	10.0 (8.9)	10.7
Ever smoked heroin	(*N* = 139)	(*N* = 120)	(*N* = 70)
Yes	96.8	97.0	95.3
No	3.2	3.0	4.7
Mean value (SD)	11.1 (7.4)	11.0 (7.4)	10.7 (6.7)

Table 1 indicates that almost half of the respondents in the introductory interview (T1) were recruited in Frankfurt's two drug consumption rooms (46.1%). Slightly less than one-third (30.9%) are from Berlin and 11.5% from Dortmund. About 1 in 20 survey participants was interviewed in Bielefeld (5.5%) and Hamburg (6.1%), respectively.

The respondents are predominantly male (77.0%). Whereas T2 shows no change in the male-female ratio compared to T1, the percentage of male clients at T3 is slightly increased (79.8%).

The survey participants' average age at T1 is 34.7 years. The average age at T2 and T3 is only slightly lower.

The question of how long the participants have been using opiates is of particular interest in this survey. While it can be assumed that long-term opiate use leads to habituated patterns of use that complicate changing the method of administration:

Table 1 indicates that the survey participants have been using heroin for an average of 13.3 years (range 1–41 years).

Almost one-fifth have been using heroin for 1 to 5 years, another 24.8% for 6 to 10 years. One-fifth reported having used heroin for 11 to 15 years and 16 to 20 years, respectively, while 15.2% reported having used opiates for more than 20 years. The respective percentages do not vary significantly between the individual stages.

Intravenous heroin use is very common among the survey participants. There is data available for 117 of the 165 respondents (70.9%) regarding the length of intravenous drug use, which has been practised for an average of 10.4 years.

The majority of persons who received a ‘SMOKE-IT!’ pack are already familiar with this method of administration.

Table 2 indicates that slightly more than two-thirds of the respondents (65.0%) injected heroin over the previous month. This method of administration is considerably more common in men (68.0%) than in women (55.3%). When differentiating by age, it is noticeable that intravenous use is more widespread in younger heroin users (age 19–29 years), accounting for 70.2%, than in those over age of 29 years.

**Table 2:** Method of heroin administration during the last 30 days before the interview (stage T1)

	**Sex**		**Age (years)**			**Total**
	**M**	**F**	**19–29**	**30–39**	**40+**	
IV heroin use last month	(*N* = 125)	(*N* = 38)	(*N* = 47)	(*N* = 66)	(*N* = 50)	(*N* = 163)
Yes	68.0%	55.3%	70.2%	62.1%	64.0%	65.0%
No	32.0%	44.7%	29.8%	37.9%	36.0%	35.0%
Times of IV heroin use per day	(*N* = 80)	(*N* = 19)	(*N* = 30)	(*N* = 39)	(*N* = 30)	(*N* = 99)
Mean value (SD)	3.2 (3.6)	4.1 (2.8)	3.6 (2.8)	3.6 (4.7)	2.8 (1.7)	3.4 (3.5)
Median	2.5	3.5	3.25	2.0	2.75	3.0
Smoked heroin last month	(*N* = 120)	(*N* = 38)	(*N* = 47)	(*N* = 64)	(*N* = 47)	(*N* = 158)
Yes	84.2%	76.3%	85.1%	79.7%	83.0%	82.3%
No	15.8%	23.7%	14.9%	20.3%	17.0%	17.7%
Smoked heroin last month	(*N* = 101)	(*N* = 29)	(*N* = 40)	(*N* = 51)	(*N* = 39)	(*N* = 130)
Less than once a week	24.8%	17.2%	32.5%	23.5%	12.8%	23.1%
Regularly, once a week	10.9%	10.3%	15.0%	9.8%	7.7%	10.8%
Regularly, several times a week	19.8%	24.1%	15.0%	21.6%	25.6%	20.8%
Regularly, at least once a day	44.6%	48.3%	37.5%	45.1%	53.8%	45.4%

Those respondents who reported injecting heroin practise this method of administration at an average of 3.4 times per day. The median, which refers to the mean value when arranging the survey participants' statements by size, is slightly lower, amounting to 3.0 injections. Very interesting differences can be seen when evaluating the data by gender. While men reported an average of 3.2 injections per day, women indicated 4.1. More intensive intravenous use among female heroin users is also confirmed in view of the median.

Among the survey participants, 82.3% reported having practised smoking heroin over the past 30 days. Smoking heroin is more prevalent among men (84.2%) than women (76.3%).

When asked about the frequency of smoking heroin, 23.1% of those respondents who used heroin that way over the past month reported choosing this method of administration less than once a week. Another 10.8% smoke heroin regularly once a week, and one-fifth (20.8%) regularly several times a week. Nearly half of the respondents (45.4%) smoke heroin regularly at least once a day, with use among women being more intensive. Almost three-fourths (72.4%) smoke heroin at least several times a week. The corresponding percentage among men is eight percentage points lower. The attractiveness of smoking heroin appears to increase steadily with the users' age. While 37.5% of those aged 19–29 years reported smoking heroin, this figure rises to 45.1% in the next older group (age 30–39 years). This relatively high percentage increases further when focusing on the oldest survey participants, 53.8% of whom smoke heroin on a daily basis.

### Two-thirds of the sample (65.3%) used the ‘SMOKE-IT!’ foils to smoke heroin instead of injecting

With two exceptions (Frankfurt - ‘La Strada’ and Hamburg ‘Ragazza’), average household smoking foils which you can buy in the supermarket had already been available at consumption rooms before the survey was conducted. After having used the ‘SMOKE-IT!’ foils, the survey participants were asked to indicate which type of smoking foil they prefer. The vast majority favoured the foils of the ‘SMOKE-IT!’ pack (85.5%). This approval is higher among female heroin users (88.9%) than male ones (81.0%). The percentage of ‘SMOKE-IT!’ supporters in the oldest group of respondents (84.6%) is a more three percentage points higher than in the other two age groups.

Intravenous heroin use poses a particular threat to the health of those who practise this method of administration, which is associated with serious consequences including vascular damage, venous disorders and risk of overdose as well as transmission of diseases such as hepatitis and HIV/AIDS. One of the survey's primary goals was therefore to reduce intravenous use among the participating heroin users.

The bottom row in Table 3 shows that two-thirds of the sample (65.3%) used the ‘SMOKE-IT!’ foils to smoke heroin instead of injecting it. This seems to be the post striking feature as it is a personal decision not to inject but to smoke heroin. The question being asked was ‘Please rethink the period between the last questionnaire (T1) until today: Did you smoke from foil instead of injecting?’

**Table 3:** Use and rating of ‘SMOKE-IT!’ foils

	**Sex**		**Age (years)**			**Total**
	**M**	**F**	**19–29**	**30–39**	**40+**	
Use of ‘SMOKE-IT!’ foils	(*N* = 109)	(*N* = 32)	(*N* = 43)	(*N* = 59)	(*N* = 39)	(*N* = 141)
Yes	87.2%	71.9%	83.7%	83.1%	84.6%	83.7%
No	12.8%	28.1%	16.3%	16.9%	15.4%	16.3%
Place of using the foils	(*N* = 95)	(*N* = 23)	(*N* = 36)	(*N* = 49)	(*N* = 33)	(*N* = 118)
Within the consumption room	44.2%	56.5%	50.0%	46.9%	42.4%	46.6%
Outside the consumption room	55.8%	43.5%	50.0%	53.1%	57.6%	53.4%
Preference of ‘SMOKE-IT!’ foils	(*N* = 79)	(*N* = 18)	(*N* = 28)	(*N* = 43)	(*N* = 26)	(*N* = 97)
Yes	81.0%	88.9%	82.1%	81.4%	84.6%	82.5%
No	19.0%	11.1%	17.9%	18.6%	15.4%	17.5%
Smoked foil instead of injecting	(*N* = 76)	(*N* = 25)	(*N* = 31)	(*N* = 46)	(*N* = 24)	(*N* = 101)
Yes	71.1%	48.0%	71.0%	60.9%	66.7%	65.3%
No	28.9%	52.0%	29.0%	39.1%	33.3%	34.7%

There are, however, significant gender-specific differences, which cannot explained within this survey. While 71.1% of men indicated smoking heroin instead of injecting it because they were given ‘SMOKE-IT!’ foils, only 48% of women reported having done so.

The differences in percentage between the individual age groups are less distinct. While 71.0% of those aged 19–29 years and 66.7% of those over age 39 years reported reduced intravenous use as a result of the ‘SMOKE-IT!’ foils, there are only 60.9% in the middle age group (age 30–39 years).

### Curiosity and reduced risks of overdose and infections - the main reasons to change the way of drug use

At the end of the T2 interview, the survey participants were asked to indicate why they smoke heroin with the new foil. Almost six in ten (58.9%) said that this method of administration was healthier than injecting. Women account for a larger percentage (66.7%) than men (56.8%) in this case. The level of agreement with this statement additionally increases with age.

Almost half of the respondents (49.1%) cited curiosity as the reason for smoking off foil, accounting for a considerable larger percentage among men (51.1%) than women (Table 4). In view of age categories, younger heroin users are particularly curious about smoking off foil (62.5%). The corresponding percentages among the older age groups are up to 20 percentage points lower.

**Table 4:** Reasons for smoking heroin

	**Sex**		**Age (years)**			**Total**
	**M**	**F**	**19–29**	**30–39**	**40+**	
	**(*N*** **= 88)**	**(*N*** **= 24)**	**(*N*** **= 32)**	**(*N*** **= 50)**	**(*N*** **= 30)**	**(*N*** **= 112)**
Curiosity	51.1%	41.7%	62.5%	42.0%	46.7%	49.1%
Healthier than injection	56.8%	66.7%	53.1%	60.0%	63.3%	58.9%
Lower risk of HIV and hepatitis	34.1%	41.7%	34.4%	40.0%	30.0%	35.7%
Lower costs	9.1%	4.2%	18.8%	4.0%	3.3%	8.0%
Quicker alleviation of withdrawal symptoms	15.9%	8.3%	12.5%	18.0%	10.0%	14.3%
Veins need a break	30.7%	29.2%	28.1%	22.0%	46.7%	30.4%
No sterile syringes available	4.5%	4.2%	6.3%	6.0%	.0%	4.5%
Avoidance of overdose	36.4%	25.0%	46.9%	28.0%	30.0%	33.9%
Recommended by others	18.2%	20.8%	28.1%	12.0%	20.0%	18.8%

For about one-third of the interviewed consumption room visitors (35.7%), the reduced risk of infection with diseases such as hepatitis and/or HIV/AIDS was a particularly significant factor. This reason was given by more women (41.7%) than men (34.1%). It is also noticeable that agreement with this item is stronger in the middle age group (40.0%) than among the youngest (34.4%) and oldest (30.0%) survey participants.

One-third of the respondents use smoking foils to avoid the danger of an overdose, with the male percentage (36.4%) being 11 percentage points higher than the female one. The levels of agreement with this reason are especially interesting in the youngest group of respondents. Almost half of them (46.9%) smoke heroin using foil for fear of overdose. In the two other age groups, this item is named by not even one-third.

There are also 30.4% who named the need to give their veins a break as a reason for using foil to smoke heroin. In terms of age groups, the respondents over age 39 years account for a higher-than-average percentage among those who said needing to give their veins a break was a major reason for smoking heroin. It can be assumed that many of the older consumption room visitors have been injecting for many years and have thereby damaged a significant part of externally accessible blood vessels, so it is not surprising that many of them reported their ‘veins need a break’.

### Drug users would also be willing to pay for foil - if they are available

Stage T3 was started after a minimum period of 30 days following self-completion of the preceding stage T2. At the beginning, the participants were asked if they had used the ‘SMOKE-IT!’ foils since T2. Table 5 shows that, with a few exceptions, this was the case (87.6%). While there were more men (91.5%) than women (72.2%) who reported using the foils, no significant differences were found between age groups.

**Table 5:** Use and rating of ‘SMOKE-IT!’ foils

	**Sex**		**Age (years)**			**Total**
	**M**	**F**	**19–29**	**30–39**	**40+**	
Use of ‘SMOKE-IT!’ foils since T2	(*N* = 71)	(*N* = 18)	(*N* = 22	(*N* = 34)	(*N* = 20)	(*N* = 89)
Yes	91.5%	72.2%	88.5%	85.4%	90.9%	87.6%
No	8.5%	27.8%	11.5%	14.6%	9.1%	12.4%
Frequency of smoking off foil	(*N* = 64)	(*N* = 12)	(*N* = 22)	(*N* = 34)	(*N* = 20)	(*N* = 76)
Less than once a week	25.0%	25.0%	18.2%	29.4%	25.0%	25.0%
Regularly, once a week	34.4%	16.7%	31.8%	29.4%	35.0%	31.6%
Regularly, several times a week	12.5%	.0%	18.2%	2.9%	15.0%	10.5%
Regularly, at least once a day	28.1%	58.3%	31.8%	38.2%	25.0%	32.9%
Use of other foils	(*N* = 71)	(*N* = 18)	(*N* = 26)	(*N* = 41)	(*N* = 22)	(*N* = 89)
Yes	42.3%	50.0%	46.2%	41.5%	45.5%	43.8%
No	57.7%	50.0%	53.8%	58.5%	54.5%	56.2%
Percentage of smoking in overall use	(*N* = 62)	(*N* = 16)	(*N* = 26)	(*N* = 35)	(*N* = 17)	(*N* = 78)
0%	12.9%	50.0%	19.2%	25.7%	11.8%	20.5%
1% to 25%	35.5%	18.8%	38.5%	31.4%	23.5%	32.1%
26% to 75%	17.7%	6.3%	15.4%	11.4%	23.5%	15.4%
76% to 99%	6.5%	.0%	3.8%	2.9%	11.8%	5.1%
100%	27.4%	25.0%	23.1%	28.6%	29.4%	26.9%
Use of “SMOKE-IT!” foils in the future	(*N* = 69)	(*N* = 18)	(*N* = 25)	(*N* = 40)	(*N* = 22)	(*N* = 87)
Yes	89.9%	44.4%	84.0%	77.5%	81.8%	80.5%
No	10.1%	55.6%	16.0%	22.5%	18.2%	19.5%
Willingness to pay for the foils	(*N* = 70)	(*N* = 18)	(*N* = 26)	(*N* = 40)	(*N* = 21)	(*N* = 87)
Yes	65.7%	29.4%	57.7%	50.0%	76.2%	58.6%
No	34.3%	70.6%	42.3%	50.0%	23.8%	41.4%
*Smoked heroin instead of injecting*	*(N = 71)*	*(N = 18)*	*(N = 26)*	*(N = 41)*	*(N = 21)*	*(N = 89)*
*Yes*	*54.9%*	*44.4%*	*53.8%*	*46.3%*	*63.6%*	*52.8%*
*No*	*45.1%*	*55.6%*	*46.2%*	*53.7%*	*36.4%*	*47.2%*

Slightly less than 60% of the participants would also be willing to pay for foil, with men again accounting for a significantly higher percentage (65.7%) than women (29.4%). There are also differences in the response patterns as far as age is concerned. While only slightly more than half of those aged 19–39 years indicated being willing to pay for foil, there are more than three-fourths (76.2%) among those over age 39 years.

When asked about a price for 10 ‘SMOKE-IT!’ foils, slightly less than one-third indicated that it should not be higher than 49 Eurocents. Another fourth would be willing to pay 50 Eurocents, while 40.4% said a price of up to 1 Euro would still be appropriate. Only two of the 52 consumption room users who replied to this question would find a price higher than 1 Euro acceptable (3.8%).

‘SMOKE-IT!’ foil can contribute to reducing intravenous use in a considerable portion of consumption room users. Slightly more than half of the respondents indicated having smoked off foil instead of injecting, with a slightly higher percentage in men (54.9%) than in women (44.4%). When distinguishing between survey participants by age, the older respondents in particular reported having changed their method of administration (63.6%). In the youngest age group, the corresponding percentage is ten percentage points lower. The lowest effect can be seen in those aged 30–39 years, with 46.3% inhaling heroin rather than injecting it.

### Conclusions and recommendations

The survey results demonstrate that the patterns of heroin users can be influenced by a mixture of new, high-quality prevention tools (foils pre-cut, uncoated, thicker and thus more resistant to tearing) and a target-group-specific approach. Similarly to the present survey, previous studies^b^ have also shown that, despite their current drug use, drug users are highly interested in preserving their health and are willing to accept measures and/or information with risk-reducing contents and objectives.

It became clear that it requires professionalism to address safer use issues during the daily routine of a drug consumption room and other drug services at the right time or at all. Some users received information about the new foils while they were waiting to enter the consumption room. A new medium enables workers to address use patterns and risks (infection, overdose) in an entirely new way. As a result, the new foil - as a new medium for arousing interest - provides new ways of approaching the users. The new foils provide an opportunity to once again address ‘old issues’ such as giving veins a break/care with a new, convincing service. Ultimately, new drug use equipment not only makes it possible to renew prevention messages or convey them for the first time, but also provides the opportunity to approach users who have so far not been reached as well as those with whom contact was lost.

It is therefore recommended that facilities which have so far exclusively offered syringe-exchange services expand their range of services to include informational literature as flyers, postcards, posters and smoking foils.

If possible, smoking foils and drug use equipment for intravenous administration should be provided free of charge. Although the survey results show that a fairly high percentage of drug users would be willing to purchase the ‘SMOKE-IT!’ foils, free-of-charge distribution is certainly preferable in order to encourage their use. In order to call the attention of the users in the facilities to this new harm-reduction service, the novelty and high quality of the ‘SMOKE-IT!’ foils should be particularly emphasised and addressed.

Special theme weeks or months had already made it possible in the past to call the attention of drug users to certain information and subjects. It is therefore recommended that a ‘SMOKE-IT! week’ be offered, accompanied by various activities and measures:

• Video tutorial on safer use and especially on inhalative use.

• How-to-smoke training courses, collective pipe and tube building (even if many users have previous experience with inhalative use, collective tube building courses or safer smoking training courses could help arouse their interest).

• Offering ‘SMOKE-IT!’ packs. The present survey demonstrated that the provision of complete packs (‘SMOKE-IT!’ packs) was also a sign of appreciation, which aroused the interest of the users of the facility in the new service.

• The utilisation level of the new service can be increased by providing informational literature that can be kept by the users, such as flyers, cards with photos of the correct smoking technique and how to make a tube/pipe,^c^ etc. The survey shows that a high percentage of those who were given ‘SMOKE-IT!’ packs also used them. Putting up posters as ‘eye-catchers’ can also raise awareness for the new service.

In view of the fact that many drug users avoid transporting drug use equipment, particularly drug consumption rooms and other low threshold services for drug users, are encouraged to support the users by offering them personalised storage space for drug use equipment.

In Germany, in 2012 about 400,000 boxes with different intravenous drug use kits are sold *via* vending machines [7]. In order to ensure the anonymous availability of smoking equipment at night, at the weekend and on public holidays, ‘SMOKE-IT!’ packs could be added to the range of products available from vending machines.

In order to initiate a discussion about the harm-reducing effects of inhalative use, facilities could develop their own individual special programmes, such as a breakfast involving a discussion about the advantages and disadvantages of inhalative use. Based on experience from on-site work, it becomes apparent that such measures can only be successful if they are very practical and their utilisation does not involve much effort for the users. Additionally, such programmes always require the commitment and motivation of the workers.

Future studies that can best add to our current understanding need to take more deeply into account cross-sectional dimensions like gender, age and ethnic and socio-economic background of the users. It should be discussed whether a long-term sustainable change of consumption patterns can be achieved and how these patterns can be stabilised over time and what kind of additional education is needed? Next step for evaluations could be, on the one hand, the interrelationship between purity/quality of heroin and initiation of smoking, as the degree of purity of heroin varies considerably all over Germany. On the other hand, Smoke-It! seems to be a promising project for a peer driven intervention, meaning that more heroin smokers would, apart from existing videos, educate people who inject drugs.

Major research funders might appropriately encourage such work in this field, if issues of cost-effectiveness are more deeply focused. However, the costs compared to injecting are as follows in Germany:

Safer inhalative drug use:

• 1 foil 0.4 Eurocent.

• 1 foil to make a pipe/tube 0.4 Eurocent.

So the total costs are 0.8 Eurocent (it should be noted that the SMOKE IT foil, especially produced to smoke heroin, can be used several times without any risks). Contrary to household foils, they are thicker and easier to smoothen again. Safer injection drug use: the costs of a complete safer use equipment with one syringe, one needle, one water ampoule, one stericup (spoon for single use), one sterifilt (filter for single use), one bag of vitamin C are about 50 Eurocents. These costs are at least five times more expensive as the equipment for inhalative use. It should be noted that only a single use is recommended.

## Endnotes

^a^UNODC.org; World Drug Report.

^b^TEST IT.

^c^Available from Deutsche AIDS-Hilfe in 2013 free of charge.

## Competing interests

The survey and the evaluation was funded by Deutsche Aids-Hilfe e.V.; Heino Stöver received no financial support for the work, which was undertaken as an extension to his general duties.

## Authors’ contributions

DS conceived the survey. HS had full responsibility for its operational management and data collection. DS and HS collaborated equally with drafting the paper. Both authors read and approved the final manuscript.
